# Genotype Delimitation in the Nod-Independent Model Legume *Aeschynomene evenia*

**DOI:** 10.1371/journal.pone.0063836

**Published:** 2013-05-23

**Authors:** Jean-François Arrighi, Fabienne Cartieaux, Clémence Chaintreuil, Spencer Brown, Marc Boursot, Eric Giraud

**Affiliations:** 1 IRD, Laboratoire des Symbioses Tropicales et Méditerranéennes, UMR IRD/SupAgro/INRA/UM2/CIRAD, Campus International de Baillarguet, Montpellier, France; 2 Centre national de la recherche scientifique, IBiSA Imagerie Gif et Imagif BioCell, Institut des Sciences du Végétal, UPR 2355, Gif-sur-Yvette, France; Cankiri Karatekin University, Turkey

## Abstract

Research on the nitrogen-fixing symbiosis has been so far focused on two model legumes, *Medicago truncatula* and *Lotus japonicus*, which use a sophisticated infection process involving infection thread formation. However, in 25% of the legumes, the bacterial entry occurs more simply in an intercellular fashion. Among them, some semi-aquatic *Aeschynomene* species present the distinctive feature to form nitrogen-fixing nodules on both roots and stems following elicitation by photosynthetic bradyrhizobia that do not produce Nod factors. This interaction is believed to represent a living testimony of the ancestral state of the rhizobium-legume symbiosis. To decipher the molecular mechanisms of this unique Nod-independent nitrogen-fixing symbiosis, we previously identified *A. evenia* C. Wright as an appropriate model legume, because it displays all the requisites for molecular and genetic approaches. To advance the use of this new model legume species, here we characterized the intraspecific diversity found in *A. evenia*. For this, the accessions available in germplasm banks were collected and subjected to morphological investigations, genotyping with RAPD and SSR markers, molecular phylogenies using ITS and single nuclear gene sequences, and cross-compatibility tests. These combined analyses revealed an important intraspecific differentiation that led us to propose a new taxonomic classification for *A. evenia* comprising two subspecies and four varieties. The *A. evenia* ssp. *evenia* contains var. *evenia* and var. *pauciciliata* whereas *A. evenia* ssp. *serrulata* comprises var. *serrulata* and var. *major*. This study provides information to exploit efficiently the diversity encountered in *A. evenia* and proposes subsp. *evenia* as the most appropriate subspecies for future projects aimed at identifying plant determinants of the Nod-independent symbiotic process.

## Introduction

The pantropical genus *Aeschynomene* belongs to the Dalbergieae tribe, an important group within the papilionoid legumes that is represented by peanut (*Arachis hypogaea*), the second most important crop legume. This genus includes approximately 150 species, one half of them from the new world, mainly South and Central America from where the genus originates, the other half found across the tropical regions of Africa, South-East Asia, Australia and the Pacific Islands [1]. The genus *Aeschynomene* comprises both herbaceous and shrubby species, annuals and perennials. Several species (*A. aspera*, *A. afraspera*, *A. nilotica*) that are profusely stem-nodulated are used as green manure for rice production due to their high nitrogen fixation rates [2] or as forage legumes (*A. americana*, *A. villosa*, *A. histrix*, *A falcata* and *A. evenia*) since they represent a good complement in proteins for cattle nutrition [3], [4]. Half of the *Aeschynomene* species are rather xeric and are found in savannas or dry forests. The other half is composed of hydrophyte species growing in marshes, rice fields, waterlogged meadows, along stream and river banks [1].

*Aeschynomene* species form nitrogen-fixing nodules in association with *Bradyrhizobium* strains. They display an infection process similar to the one described in *Arachis hypogaea* and other species belonging to the Dalbergioid clade [5], [6], [7]. The mode of infection usually referred as “crack entry” is characterized by the entry of the bacteria that occurs intercellularly between epidermal cells at the emergence of lateral roots. The nodule originates then from the successive divisions of only one or a few infected cortical cells, also called founder cells. As similar infection process called “direct entry” is also found in the Genistoid clade. This intercellular infection pathway that is supposed to occur in 25% of legumes species consequently differs from the infection process described for the model legumes *Medicago truncatula* and *Lotus japonicus* for which bacterial entry is controlled by the formation of infection threads and implies the distant induction of a nodular primordium [8], [9].

The initial interest of studying the *Aeschynomene* relies on the fact that 22 semi-aquatic species have the capacity to form nitrogen-fixing nodules on both roots and stems. This very unusual behavior among legumes is only shared with a very few species of the genera *Sesbania*, *Neptunia* and *Discolobium* [10]. In addition, some bradyrhizobia isolated from *Aeschynomene* stem nodules exhibit a property uncommon among other rhizobia of developing a photosynthetic system [11], [12]. It has been shown that the photosynthetic activity of *Bradyrhizobium* plays a key role during stem nodulation by directly furnishing energy to the bacterium that can be used for biological nitrogen fixation [13], [14]. More recently, some photosynthetic *Bradyrhizobium* strains were shown to be unique because they lack the canonical *nodABC* genes required for the synthesis of Nod factors whereas they maintain the ability to elicit efficient nodules [15]. This revealed the existence of a Nod-independent symbiotic process. At present, this original symbiotic interaction has been found only in 11 semi-aquatic *Aeschynomene* species (C. Chaintreuil, unpublished data).

This Nod-independent process is believed to represent a living testimony of the ancestral state of the rhizobium-legume symbiosis [7], [16]. Its unravelling should therefore shed new light on the evolution of rhizobium-legume symbiosis and could have important agronomic implications, notably to identify new strategies to engineer nitrogen-fixing nodules in cereals [17], [18]. To decipher the mechanisms of this new symbiotic process, the identification of an appropriate diploid species allowing the development of genetic approaches is required. For this, we previously explored the diversity of the 11 Nod-independent *Aeschynomene* spp. Among them, *A. evenia* C. Wright appears the most promising because it displays all the characteristics required for genetic and molecular analysis [19]. Besides its diploid character and relatively small genome (2n = 20, 460 Mb/1C), it is a short-perennial and autogamous species. *A. evenia* is nodulated by the well-characterised photosynthetic *Bradyrhizobium* sp. strain ORS278 and is efficiently transformed by *Agrobacterium rhizogenes*. Furthermore, *A. evenia* is genetically homozygous but polymorphism has been evidenced between accessions that can be successfully hybridized.

In order to develop genetic and mapping approaches, a good knowledge of the phenotypic and genotypic diversity encountered in *A. evenia* is necessary. But this has been only described at a botanical level. In this study, we explore the intraspecific diversity of *A. evenia* using the accessions available in germplasm collections and by combining morphometric, genetic, molecular and crossing compatibility approaches. This allowed us to evidence intraspecific delimitations and to propose a new nomenclature for the genotypes encountered in *A. evenia*.

## Results and Discussion

### Phenotypic and Genotypic Polymorphism in the *A. evenia* Species

To investigate the intraspecific diversity in *A. evenia*, we thoroughly characterized a set of twenty seven accessions procured from USDA (USA), CIAT (Colombia), AusPGRIS (Australia) and the Senegal herbarium (University Cheikh Anta Diop, Dakar) (Table 1). They represent almost all the accessions available in germplasm banks. These accessions originate from regions of the world where this species naturally grows: mainly Brazil but also Mexico, Argentina, Venezuela, Malawi and Senegal. The cultivar IRFL6945, which served as reference in the previous study [19], was selected in Florida (USA) for use as forage legume but is probably not native of this region [20], [21].

**Table 1:** Accessions of A*eschynomene* used in this study, origin and characteristics.

Species/Group	Subgroup	Accession	2C DNA content (pg)	ITS	Origin	Seed bank^b^
*A. evenia* group *serrulata*	*Goias*	CIAT 7560^a^	0.99±0.01	simple	Goias, Brazil	CIAT
		CIAT 7562	0.97±0.01	simple	Goias, Brazil	CIAT
		CIAT 7571	–	–	Bahia, Brazil	CIAT
		CIAT 8223	–	–	Bahia, Brazil	CIAT
		CIAT 8228	–	–	Bahia, Brazil	CIAT
		CIAT 8936	–	–	Bahia, Brazil	CIAT
		IRFL 6945	0.95±0.01	double	Florida, USA	USDA
	*Alagoas*	CIAT 8242^a^	0.95±0.01	double	Alagoas, Brazil	CIAT
		CIAT 8244	0.94±0.01	simple	Pernambuco, Brazil	CIAT
		CIAT 18989	0.94±0.01	simple	Bolivar, Venezuela	CIAT
*A. evenia* group *evenia*	*Bahia*	CIAT 8232^a^	0.82±0.01	simple	Bahia, Brazil	CIAT
		CIAT 8245	0.86±0.01	simple	Pernambuco, Brazil	CIAT
		CIAT 8258	–	–	Alagoas, Brazil	CIAT
		CIAT 8261	0.83±0.01	simple	Sergipe, Brazil	CIAT
		CIAT 8938	0.85±0.02	simple	Bahia, Brazil	CIAT
		CIAT 8944	–	–	Bahia, Brazil	CIAT
		CIAT 9539	–	–	Sao Paulo, Brazil	CIAT
	*Paraiba*	CIAT 8251	0.87±0.01	simple	R.Gde Do Norte, Brazil	CIAT
		CIAT 8254^a^	0.83±0.01	simple	Paraiba, Brazil	CIAT
		CIAT 8426	0.86±0.01	simple	Pernambuco, Brazil	CIAT
		CPI 43192	–	simple	Bahia, Brazil	AusPGRIS
	*Mbao*	CIAT 22700^a^	0.84±0.01	simple	Mbao, Senegal	CIAT
		CIAT 22838	0.85±0.02	double	Liwonde, Malawi	CIAT
		STM 29-bis	0.86±0.01	simple	Kaolack, Senegal	UCAD
		STM 45	0.85±0.01	simple	Niayes, Senegal	UCAD
	*Salta*	ATF 3087^a^	0.83±0.01	simple	Salta, Argentina	AusPGRIS
		CPI 90919	0.85±0.02	simple	La Cruz, Mexico	AusPGRIS
*A. denticulata*		IRRI 13003	1.29±0.03	simple	Brazil	IRRI
*A. ciliata*		IRRI 13078	1.08±0.02	simple	Colombia	IRRI

a accession considered as representative of the group and whose geographical localisation has been used to name it.

b CIAT, Centro International de Agricultura Tropical (http://isa.ciat.cgiar.org); USDA, United States Department of Agriculture (http://www.ars-grin.gov/npgs); AusPGRIS, Australian Plant Genetic Ressource Information Service (http://www2.dpi.qld.gov.au/extra/asp/auspgris); IRRI, International Rice Research Institute (www.irri.org); LSTM, Laboratoire des Symbioses Tropicales et Méditerranéennes (www.mpl.ird.fr/lstm).

- : not determined.

#### Morphological characterization

**Figure 1:**
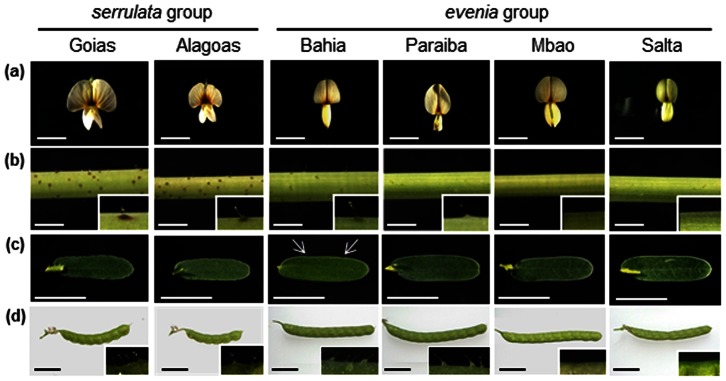
Phenotypic polymorphism in *A. evenia* accessions. Morphological variations in *A. evenia* which distinguish the different *evenia* and *serrulata* groups and subgroups based on flower (a), stems with a zoom on thrichomes (b), leaves with arrows pointing to denticulations terminated with cilia (c) and pods with a zoom on cilia (d). Scale bars = 6 mm.

This set of accessions was grown in greenhouse conditions and evaluated for morphological characters. As previously described in Arrighi et al. [19], two major phenotypic groups could be differentiated. They correspond to the two described botanical varieties: var. *evenia* and var. *serrulata* [1]. The *evenia* group is characterized by mostly glabrate stems, tender-green leaves with leaflets predominantly entire and flowers with an elliptic standard petal (Fig. 1). The *serrulata* group is distinctive in that the stems are conspicuously covered with glandular thrichomes. Leaves are dark-green and smaller, with leaflets consistently serrulate and ciliate. Flowers are recognizable by their rounded/obcordate standard petal (Fig. 1).

However, within each main group, different sub-morphogroups could be distinguished. For convenience, they were named according the geographical location of a representative accession (Table 1). Hence, in the *serrulata* group, the subgroup Alagoas differed by the plants having flowers and pods smaller than for Goias (Fig. 1, Table S1 in File S1). Similarly, in the *evenia* group, the subgroup Bahia was distinctive in that most of the accessions presented moderately denticulate and ciliate leaflets, the subgroup Mbao displayed dark yellow anthers and the subgroup Salta formed the smallest flowers in the series (Fig. 1, Table S1 in File S1). Based on these morphological subgroups (Table1), two to three accessions for each morphotype were selected for detailed analysis (Table S1 in File S1).

#### Genotypic characterization

**Figure 2:**
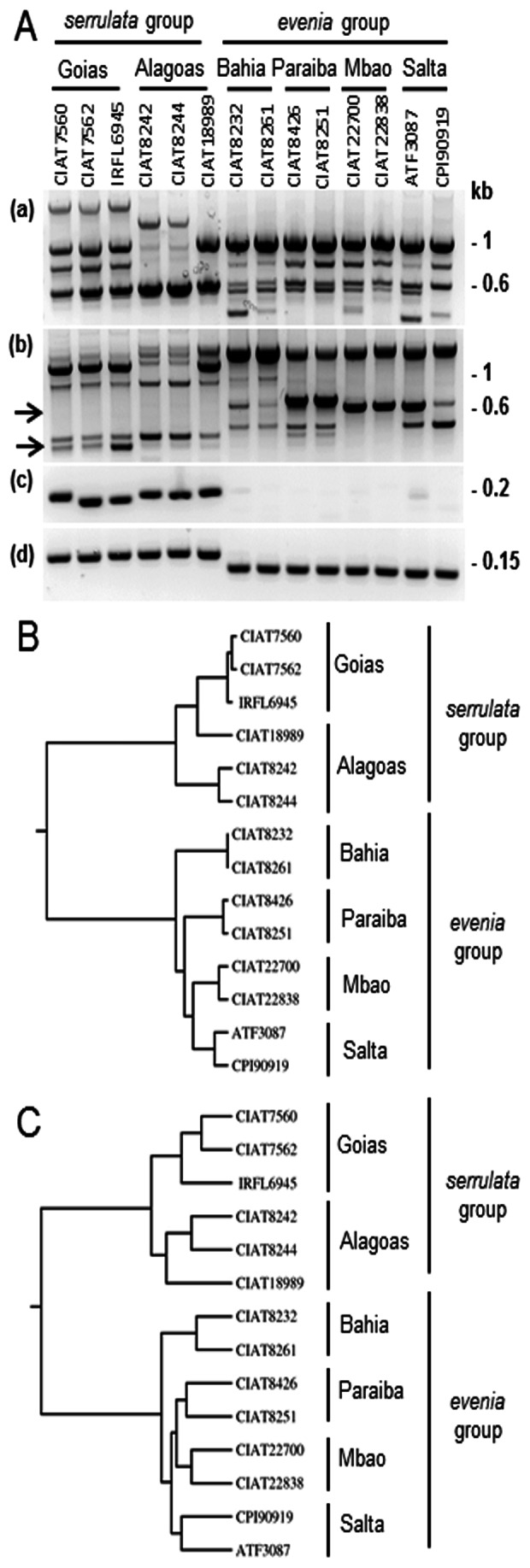
Genotypic polymorphism in *A. evenia* accessions. **A,** PCR amplification profiles obtained using RAPD and SSR markers. RAPD profiles using the OPAB7 primer (a) and the B6 primer (b) showing distinctive banding patterns for the two main groups and bands specific to certain subgroups. (c) SSR profile using the *Ai*SSR46 marker showing a group-specific amplification. (d) SSR profile using the *Ae*SSR20 marker showing a clear separation in allele size for each group: *evenia* and *serrulata*. **B,** UPGMA dendrogram obtained with the RAPD genotyping (23 primers used) for representative accessions of each subgroup. **C,** UPGMA dendrogram obtained with the SSR genotyping (82 SSR markers used) for representative accessions of each subgroup.

To assess the genetic diversity and relationships between the different morphotypes, the selected accessions were subjected to Randomly Amplified Polymorphic DNA (RAPD) analysis. Multilocus genotyping by 23 RAPD primers generated scorable amplicons with reproducible patterns. As already noticed [19], the two series displayed for most RAPD markers distinctive banding patterns (Fig. 2A–a). Interestingly, for several RAPDs, specific bands were also detected for each morphotype (Fig. 2A–b, Table S2 in File S1). The dendogram obtained delineated the two groups with clusters corresponding almost exactly to the 6 morphotypes (Fig. 2B). The similarity, calculated using the DICE coefficient, ranged from 66% to 99% within each group whereas it ranged only between 9% and 20% when comparing the *evenia* group with the *serrulata* group (Table S3 in File S1).

The accessions were also genotyped using Simple Sequence Repeats (SSR) markers. They were obtained from EST sequences available for *A. evenia* IRFL6945 (52 SSRs) and the related *A. indica* (30 SSRs) (F. Cartieaux, unpublished data). Intriguingly, 24 out of the 82 SSRs tested were amplified in only one of the two groups (Fig. 2A–c, Table S2 in File S1). In addition, 25 out of the remaining 58 SSRs presented alleles of different size ranges for the *evenia* and *serrulata* groups (Fig. 2A–d, Table S2 in File S1). Polymorphism was also found between and within each of the 6 morphotypes. It was estimated with the distance matrix using the DICE coefficient (Table S4 in File S1). Hence, intra-group polymorphism ranged from 11 to 37% whereas inter-group polymorphism rose to 64–79%. The SSR genotyping data were processed to generate a dendogram that was found to be very similar to those obtained with the morphological and RAPD analyses (Fig. 2C).

The whole collection was tested with a subset of discriminating RAPD and SSR markers. We found that all the accessions belonging to the same morphotype displayed similar RAPD and SSR profiles (data not shown). This indicated a consistent concordance between morphotypes and genotypes, and so, the relevance of the selected phenotypic criteria to generate groups and subgroups.

#### Genomic DNA quantification

To get another insight into the differentiation in *A. evenia*, accessions of each group were also analyzed to determine their genome size by flow cytometry (Table 1). The *serrulata* cultivar IRFL6945 was previously found to contain a 0.95 pg/2C genome [19]. Accordingly, all the *serrulata* accessions tested exhibited similar DNA content comprised between 0.94 and 0.99 pg/2C. The *evenia* accessions differed by a smaller genome size of 0.82–0.87 pg/2C. Thus, although small variations are also present within each variety, it is worth noting the range of genome sizes are distinct between the two groups with a 12% genome size variation.

### Molecular Analysis of the Intraspecific Differentiation

On the basis of congruent delineations using morphological, RAPD and SSR data, the representative accessions for each of the 6 clusters were further characterized for the intraspecific differentiation. They were used for the sequencing of nuclear genes and the realization of molecular phylogenetic analyses. *A. denticulata* and *A. ciliata* that are the two closest relative diploid species to *A. evenia* [1], [19] were added to this study in order to root the phylogenic trees obtained.

#### Analysis of single nuclear gene sequences

**Figure 3:**
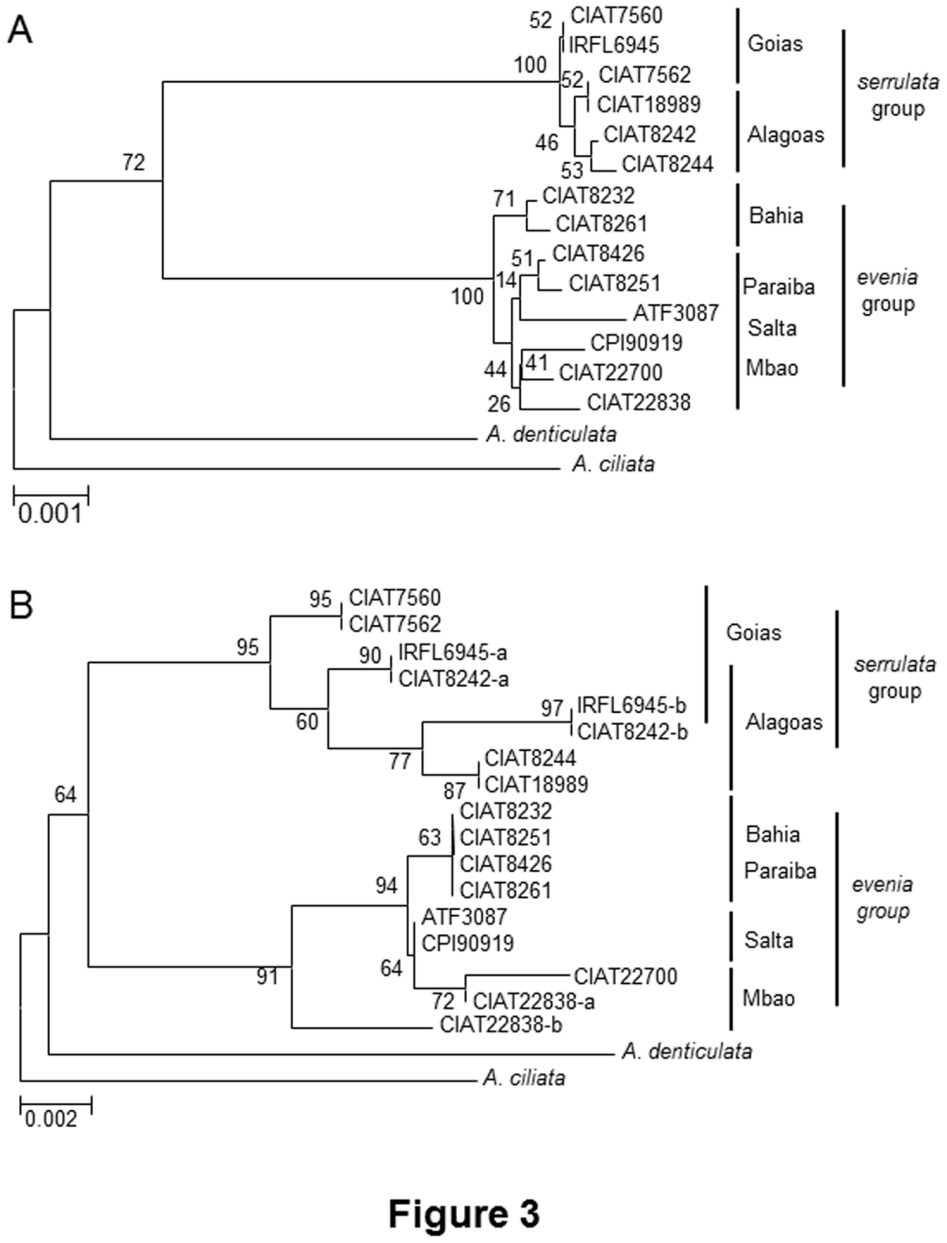
Phylogenetic trees based on nuclear gene sequences. **A,** Phylogeny of 6 concatenated single gene fragments (the cyclophylin 1 (*CYP1*), the eukaryotic translation intitiation factor 1A (*eIF1a)*, the Sucrose Synthase, the translation factor *SUI1*, a gene coding for a putative 2OG-Fe(II) oxygenase, and a legume-specific gene homolog to Glyma07g16420 and Glyma18g37410 identified in *Glycine max*). **B,** Phylogeny of the ITS sequence. Representative accessions of each subgroup of *A. evenia* were characterized with *A. denticulata* and *A. ciliata* were used here as outgroups in order to root the trees. −a and −b indicate paralogous ITS sequences found in the same accession. Numbers at nodes represent bootstrap values (% of 1,000 replicates).

First, an RNA library sequenced for the cultivar IRFL6945 (F. Cartieaux, unpublished data) was mined to identify nuclear genes. Six genes in single copy in *A. evenia* were selected: the cyclophylin 1 (*CYP1*), the eukaryotic translation intitiation factor 1A (*eIF1a)*, the Sucrose synthase, the translation factor *SUI1*, a gene coding for a putative 2OG-Fe(II) oxygenase, and a legume-specific gene encoding a protein of unknown function (Tables S7 and S8 in File S1). They were amplified in two to three representative accessions of each genotype, generating for each a single sequence. In order to produce a well-resolved phylogeny, the coding sequences of the six genes were concatenated into a unique 2300-pb sequence for each genotype and subjected to maximum likelihood Bayesian analysis. The topology of the resulting tree depicted two major clusters corresponding to the *evenia* and the *serrulata* groups, and both *A. denticulata* and *A. ciliata* as outgroups (Fig. 3A). The Bahia subgroup was well-differentiated from the other *evenia* subgroups. We compared the concatenated sequences by recording single nucleotide polymorphisms (SNPs) as well as some insertions and deletions (INDELS) of tri-nucleotidic repeats at a number of positions in the coding sequences. A 27-to-30/2300 substitution rate was thus calculated between the *evenia* and *serrulata* groups whereas this rate was only of 0–2 within the *serrulata* group and 1–7 within the *evenia* group (Table S5 in File S1).

#### Analysis of ITS sequences

Similar analysis was performed using the sequence of the ITS1–5.8S rDNA gene-ITS2 region. ITS amplification and sequencing lead in certain cases to chromatograms with several double peaks. In such cases, the PCR products were cloned and sequenced separately. This occurred once in the *evenia* group (CIAT22838: two sequences differing by 1 INDEL and 6 SNP over 704 pb, corresponding to 1% divergence) and twice in the *serrulata* group (IRFL6945 and CIAT8242: two sequences differing by 1 INDEL and 5 SNPs over 704 pb, corresponding to 0.85% of divergence) (Table 1, Fig. S1). The ITS sequences obtained for each accession were used to generate a phylogram (Fig. 3B). Robust relationships (bootstrap scores above 80%) at the two nodes corresponding to the groups were found. Thus, the *evenia* ITS sequences strongly clustered together with three subgroups corresponding to Bahia/Paraiba, Mbao and Salta. Only the second ITS sequence found in the accession CIAT22838 was more distant but fell in the same clade. The second well-supported cluster corresponded to ITS sequences found in the *serrulata* group in which two subgroups corresponding to Alagoas and Goias could also be distinguished. Interestingly, the two accessions IRFL6945 and CIAT8242 that belong respectively to Goias and Alagoas presented both ITS sequences. Here again, *A. denticulata* and *A. ciliata* were more distantly related. Presence of more than one ITS sequence within individuals has already been reported for other species [22], [23]. They may correspond to pseudogenes or indicate introgressions followed by incomplete ITS sequence homogenization. However, in our case, this did not infer in the phylogenic reconstruction. It is worth noting that the maximum divergence levels corresponded to 1% within both the *serrulata* and *evenia* groups compared to the 2.15–2.55% between the two groups. Thus, the same deep split between the *evenia* and *serrulata* groups was observed, whatever the considered ITS.

All in all, the analysis of both single nuclear genes and ITS sequences evidenced an important differentiation between the *evenia* and *serrulata* groups that form sister clades. Moderate variations were also observed within each group, indicating intra-group polymorphism as revealed by the RAPD and SSR profiles.

### Hybridization Analysis of the Inter- and Intra-varietal Genetic Compatibility

In order to test whether the genetic and genomic differentiations evidenced in *A. evenia* may be accompanied with crossing incompatibilities, artificial hybridizations were performed between accessions of each group. This was facilitated by the fact that i) flowers of both varieties share the same selfing mechanism and ii) an efficient manual crossing method had been previously developed [19].

#### Inter- and intra-group crossings

Hybridizations done between the *serrulata* subgroups Goias and Alagoas, as well as in the *evenia* subgroups Bahia with Paraiba, Mbao and Salta were highly successful (Table 2). Conversely, inter-groups crossings using *serrulata* accessions as the maternal parent all failed: either the recipient flower fell down directly one day after pollination or a young pod started to develop but rapidly aborted. In contrast, using *evenia* accessions as the maternal parent lead to the development of fully developed seed-bearing pods (Table 2). In this case, the F1 seeds displayed a wrinkle integument, in contrast to the crossing parents (Fig. S2A). Use of different *serrulata* and *evenia* crossing parents produced the same results, indicating that the observations were not due to restricted incompatibilities between two specific accessions.

**Table 2:** Inter- and intra-group crossability in *A. evenia.*

Successful/total crossings		♀					
		*serrulata* group		*evenia* group			
		Goias	Alagoas	Bahia	Paraiba	Mbao	Salta
♂	Goias	15/15	10/10	8/8*	2/4*	4/4*	2/3*
	Alagoas	8/8	5/6	5/7*	**–**	2/2*	3/3*
	Bahia	0/15	0/6	5/5	**–**	4/6	**–**
	Paraiba	0/15	0/6	5/5	5/5	2/2	4/5
	Mbao	0/26	0/9	7/7	4/5	5/5	**–**
	Salta	**–**	**–**	**–**	**–**	**–**	5/5
	Ø	0/14	0/7	0/6	0/4	0/6	0/5

n/n : successful crossings leading to fully developped seed-bearing pods/total performed crossings.

Ø : emasculated recipient flowers that were not manually pollinated.

* : pods containing viable seeds but with a wrinkled integument.

- : Not tested.

#### Analysis of F1 and F2 progenies

For each kind of hybridization (intra-*serrulata*, intra-*evenia* and *evenia* × *serrulata*), one crossing was selected to further analyse the progeny (Table 3). The F1 plants were all vigorous and abundantly flourished as the parental lines. However, they greatly differed in their fertility: the intra-group hybrids readily produced seeds whereas only few seeds were collected from the inter-group F1 plants. To exclude the possibility this sterility was due to anatomical problems, the flower structure for the inter-group F1 plants was observed and compared to *evenia* and *serrulata* parental lines (Fig. S2B). Once the flower opened, indicating that pollination had normally occurred, anthers were found to face the stigma and pollen was obviously liberated as already described [19]. This excluded at first sight the possibility of a low level of fertility due to anatomical disorders. The resulting intra- and inter-group F2 progenies were also vigorous and no obvious developmental or physiological defect was observed (data not shown). SSR analysis of the F2 plants evidenced the presence of the three expected genotypes corresponding to the parental alleles and the heterozygotes (Table 3 and Fig. S2C). This suggests that despite the partial inter-group cross-incompatibility and sterility barrier observed, intergenomic exchanges between the *evenia* and *serrulata* groups remain possible. However, in the optic of developing mapping approaches, the use of parent lines belonging to the same group would appear to be preferable.

**Table 3:** Characteristics of F1 and F2 progenies from crosses in *A. evenia.*

Crossings	F1			F2		
	Seed aspect	Plant health	Fertility (seed set/2 months)	Seed aspect	Plant health	Detected genotypes*
Goias × Alagoas	WT	vigorous	200	WT	vigorous	3
Goias × Bahia	Wrinkled	vigorous	8	WT	vigorous	3
Bahia × Mbao	WT	vigorous	300	WT	vigorous	3

* : genotypes corresponding to the two parental alleles (homozygous) and to hybrids (heterozygous). For each crossing, F2 plants were genotyped with 4 AiSSR markers.

- : Not tested.

### Taxonomic Status of the Infra-specific Groups in the *A. evenia*

The polyphasic analysis conducted in this study revealed an important infraspecific differentiation in *A. evenia*, with the phenotypic, genotypic, molecular and cross-compatibility analyses being highly congruent. They evidenced the presence of two distinct major groups corresponding to the botanical varieties defined by Rudd [1]: var. *evenia* and var. *serrulata*, with four and two subgroups respectively that could be also defined.

#### Status of the botanical varieties *evenia* and *serrulata*

Our data highlighted a strong infraspecific genetic split between the *evenia* and *serrulata* groups, with the RAPD and SSR profiles being consistently distinctive. In addition, the tested SSR markers showed only a 70% inter-varietal transferability and many differed in their size range between var. *evenia* and var. *serrulata*. These results suggest these varieties are genetically divergent and that the gene flow from one to the other seems to be impeded. This view is supported by the 12% difference in genome sizes and by the difficulty to obtain fertile hybrids during manual inter-varietal crossings. This is reminiscent to what has been described in other legume species such as *Arachis hypogaea* [24], [25], *Vigna unguiculata* [26] and *Medicago truncatula* [27], [28]. In each case, advanced intraspecific differentiations lead to distinguish different subspecies, varieties and/or genotypes. For *A. hypogaea* and *V. unguiculata*, a high degree of congruence between subspecies and variety delimitation was observed on the basis of morphological and molecular characterisation [24], [25], [26]. But the situation encountered in *A. evenia* is more closely related to *M. truncatula* where the subspecies *truncatula* and *tricycla* have been shown to display different genome sizes [27], distinct genetic profiles [28] and for which inter-subspecies crossings lead to hybrids that showed a reduction in fertility. In addition, molecular phylogenies using single nuclear genes and ITS sequences revealed that *evenia* and *serrulata* groups form sister taxa, with the most closely related diploid species, *A. denticulata* and *A. ciliata*, being more distant. All these data suggest there is an ongoing speciation process occurring in *A. evenia* and therefore the two botanical varieties described should be elevated to the rank of subspecies.

#### Status of the six identified genotypes in *A. evenia*

The two proposed subspecies were also further subdivided into different well-defined subgroups based on both morphological and genotypic characters. Notably, each subgroup was characterized by specific phenotypic features along with distinctive RAPD and SSR profiles. Only the *serrulata* accession CIAT 18989 was found to have an intermediary position between the Goias and Alagoas groups based on the RAPD profiles. This suggests it could be the result of a crossing between parents belonging to these two subgroups. Polymorphism level between groups of the same subspecies was estimated to range from 19 to 37% using SSR markers and to be 0–7/2300 bases using nucleotide variations in coding sequences of six single nuclear genes. However, within each subspecies, genomic DNA content was found to be homogenous and the groups were entirely cross-compatible producing fully-fertile hybrids, thus supporting the concept of subspecies. The similar genetic and molecular profiles found within each group further indicate they correspond to six distinct genotypes. In the *serrulata* subspecies, the genetic differences are consistent enough (31–34% RAPD polymorphism and 31–37% SSR polymorphism) to classify the two genotypes as genuine varieties. For the *evenia* subspecies, it is worth noting that the genotype Bahia appears to be the more divergent, regardless of the criteria used (morphological, RAPD and SSR profiles, gene sequences) and thus displays the highest differentiation level compared to the three other genotypes (21–29% for RAPDs and 24–33% for SSRs). This suggests it could be considered as a separate variety whereas the three other genotypes are part of a second variety.

### Proposal for a New infra-specific Classification in *A. evenia*

In the light of the data obtained with this study, we propose a revision of the taxonomic classification for *A. evenia* with new genotype delimitations. Here, we recognize two subspecies: *A. evenia* ssp. *evenia* and *A. evenia* ssp. *serrulata*, each containing two varieties. They are listed below with the distinctive morphological features emphasized and the genotypes they contain (summary in Table 4).

**Table 4:** Proposition for a new classification within the species *Aeschynomene evenia* species C. Wright.

Subspecies	Varieties	Genotypes	Phenotypic characteristics	Commentary
*A. evenia* ssp. *evenia*	var. *evenia* C. Wright	Paraiba MbaoSalta	Leaflets: entire Stems: glabrous Pods:ciliate or glabrate	Typical variety (C. Wright)
	var. *pauciciliata* J.F Arrighi var. *nov.*	Bahia	Leaflets: sparcely serrulate-ciliate Stems:glabrous Pods: ciliate	Discrete presence of serrulata-like characters
*A. evenia* ssp. *serrulata*	var. *serrulata* V.E. Rudd	Alagoas	Leaflets: serrulate-ciliate Stems: glandular	Typical variety (V.E. Rudd)
	var. *major* J.F Arrighi var. nov.	Goias	Leaflets: serrulate-ciliate Stems:glandular Flower size: bigger	Germplasm IRFL6945 used as a forage legume

#### Aeschynomene evenia ssp. Evenia

Corresponds to the formerly described *A. evenia* var. *evenia* C. Wright, whose complete description can be found in Rudd [1].

Short description: Plants with a predominant primary axis or lateral branches developed in the upper part. Stems mostly glabrate or sparsely hispidulous. Leaves 16–30 foliate, leaflets predominantly entire, occasionally with few cilia. Flowers with an elliptic standard, 9–11 mm long and 4.5–7 mm wide. Pods 7–12 articulate, ciliate or glabrate with the upper margin straight and the lower margin sub-crenate. Seeds about 2–3 mm long and wide, brown.

#### Aeschynomene evenia ssp. evenia var. evenia C. Wright

Etymology: means “smooth” in reference of the entire leaflets and glabrate stems.

Description: The typical variety is characterised by leaflets entire and pods hispidulous or glabrate occasionally bearing sparse thrichomes with a red basis. Accessions of this group display mostly a primary axis with short flower-bearing lateral axes.

Type: CIAT 8254. Collected at Caico-Patos, kms: 33, Direction north-east Road Br 226, R. 110, Paraiba, Brazil, July 23, 1980, by Rainer Schultze Kraft, Lidio Coradin, Glocimar Pereira Da Silva. Accession available at the CIAT stock center.

Specimens examined: The complete description was based on the accessions CIAT 8251 (Brazil), CIAT 8254 (Brazil), CIAT8426 (Brazil), CIAT 22700 (Senegal), CIAT 22838 (Malawi), ATF3047 (Argentina) and CPI 90919 (Mexico) (Table 1).

Genotypes: Paraiba, Mbao and Salta.

#### *Aeschynomene evenia* ssp. *evenia* var. *pauciciliata* J.F. Arrighi, *var. nov*. [urn:lsid:ipni.org:names:77126198-1]

Etymology: named in reference to the discrete serrulate-ciliate character of leaflets observed in some accessions.

Description: This variety includes accessions with leaflets that are moderately denticulate and ciliate or entire. Pods are hispidulous. Plants of this group have the propensity to bear well-developed branches on the primary axis.

Type: CIAT 8232. Collected at Itabuna-Salvador, kms: 147, Direction north, “km 361” Road Br 101, entry of A Gandu, Bahia, Brazil, July 17, 1980, by Rainer Schultze Kraft, Lidio Coradin, Glocimar Pereira Da Silva. Accession available at the CIAT stock center.

Specimens examined: The complete description was based on the accessions CIAT 8232 and CIAT 8261 (Brazil) (Table 1).

Genotypes: Bahia.

Commentary: The discrete denticulate and ciliate character of leaflets of some accessions suggested to Rudd [1] they correspond to intergradations between the two formerly-defined varieties. This study revealed that, although they are genuine *evenia* accessions, they appear to form a basal taxa within the *evenia* subspecies.

#### Aeschynomene evenia ssp. Serrulata

Corresponds to the formerly described *A. evenia* var. *serrulata* Rudd, with a complete description available in Rudd [1].

Short description: Plants of this taxon are basally branched and are much more glandular in all parts than the *evenia* subspecies. Leaflets are consistently denticulate and ciliate and mostly smaller and with a darker green. Flowers are 7–10 mm-long and 5.5–9 mm-wide, and differ by a rounded-to-obcordate standard petal. Pods are 5–9 articulate, covered with glandular thrichomes, with the upper margin subentire and the lower margin crenate. Seeds about 1.8–3 mm. long and wide, brown.

#### Aeschynomene evenia ssp. serrulata var. serrulata V.E. Rudd

Etymology: named in reference to the serrulate aspect of the leaflets evoking the teeth of a saw.

Description: This is the typical variety with flowers 7–8 mm long and 5.5–6 mm wide, pods that are commonly 5–7 articulate.

Type: CIAT 8242. Collected at Maceio-Recife, Kms 53, direction Noreste, Road Br 101, Alagoas Brazil, July 21, 1980, by Rainer Schultze Kraft, Lidio Coradin, Glocimar Pereira Da Silva. Accession available at the CIAT stock center.

Specimens examined: The complete description was based on the accessions CIAT 8242 (Brazil), CIAT 8244 (Brazil) and CIAT18989 (Venezuela) (Table 1).

Genotypes: Alagoas.

Commentary: The forms with smaller flowers are described by Rudd [1] as being the most widespread. This morphotype is thus chosen as the typical variety.

#### *Aeschynomene evenia* ssp. *serrulata* var. *major* J.F. Arrighi, *var. nov*. [urn:lsid:ipni.org:names: 77126199-1]

Etymology: named in reference to the bigger size of the flowers for the accessions of this group compared to the typical variety.

Description: This variety is morphologically similar to *serrulata* but is distinguished by the general bigger size of the plants and of some organs: flowers 8–10 mm long and 7–9 mm wide, pods are mostly 6–9 articulate.

Type: CIAT 7560. Collected at Brasilia-barreiras, Kms 242, direction Noreste, Road Br 020, Alvorada Do Norte, Goais, Brazil, September 27, 1978, by Rainer Schultze Kraft, Lidio Coradin, Jose C. Silva, Glocimar Pereira Da Silva. Accession available at the CIAT stock center.

Specimens examined: The complete description was based on the accessions CIAT 7560 (Brazil), CIAT 7562 (Brazil) and IRFL 6945 (USA) (Table 1).

Genotype: Goias.

Commentary: The cultivar IRFL 6945, belonging to this group, corresponds to a breeding line selected at the IFAS, University of Florida, Fort Pierce (USA), 1997, by A.A. Kretschmer, for a use as legume forage [20], [21].

### Conclusions

The proposed taxonomic classification reflects more accurately the genetic diversity present in *A. evenia.* It now allows a fine delimitation of different genotypes: Goias, Alagoas, Bahia, Paraiba, Mbao and Salta. With the delineation of two subspecies and four varieties clustering these genotypes, it represents a solid taxonomic framework that could be enriched with future inclusion of more accessions, thus enhancing our knowledge of *A. evenia* diversity. This task will help in better understanding the diversification and dispersal process for this species that is thought to originate from South America [1] but that is also present in Africa. Such knowledge is also of importance for the judicious exploitation of both the phenotypic and genetic variability in the model legume *A. evenia*.

Interestingly, in each subspecies, varieties were shown to be entirely cross-compatible and the polymorphism levels were found to be significant. Previous work [19] allowed us to determine that the cultivar IRFL 6945 belonging to the *A. evenia* ssp. *serrulata* was suitable for molecular and genetic analysis. This present study has revealed that accessions from *A. evenia* ssp. *evenia* present the interest to have a smaller genome size (415 Mb vs 465 Mb for *A. evenia* ssp. *serrulata* where 1 pg DNA = 978 Mb). In addition, some of these accessions also displayed a shorter cycle growth accompanied with a more profuse seed set and a less branching habit. These developmental advantages can also be of interest for an efficient management of plant culture.

The knowledge acquired with this study will be of significance to perform artificial crosses and to develop genetic approaches aiming at identifying the plant determinants of the Nod-independent symbiosis. Genetic diversity will be important to build a genetic map that will serve both to map genes of interest and to assist future whole genome sequencing.

In conclusion, by unraveling the intraspecific diversity in *A. evenia* and identifying the potential advantages of using *A. evenia* ssp. *evenia* for molecular genetics, this work should benefit to the legume research community in its quest to decipher the mechanisms of the Nod-independent symbiosis.

## Materials and Methods

### Plant Material, Culture Conditions and Crossing Procedure

All the accessions of *A. evenia* used in this study, their geographic origin and their sources are listed in Table 1. Seed germination, plant culture in greenhouse and hybridizations were performed as indicated in Arrighi et al. [19].

The characterization of the 27 accessions was conducted in greenhouse in the autumn-winter seasons of 2011 and 2012. A randomized design was used with three plants per accession. Although a selected set of accessions was thoroughly characterized at the morphological and molecular levels, the remainder of the accessions was characterized in other assays in order to check the morphological, genetic and taxonomic classifications.

### Morphological Analysis

The accessions grown in greenhouse conditions were evaluated for morphological characters such as plant habit, plant height, presence or not of trichomes on the stem, leaf color, leaflet shape, flower type, flower dimensions, pod shape, pod surface, number of seeds per pod, seed size and shape.

Macroscopy images were obtained using either an optical Macroscope (Nikon AZ100, Champigny-sur-Marne, France) or a Digital Presenter UF-130ST (Samsung, Korea). Seed length was determinated using the ImageJ software (Wayne Rasband, National Institutes of Health, USA; http://imagej.nih.gov/ij).

### DNA Isolation, Amplification, Typing and Sequencing

Total genomic DNA was isolated from young leaves of plants using the CTAB extraction method [29]. Primers for RAPD markers were designed by Operon Technologies (Alameda, Calif, USA) (Table S6 A in File S1). SSR motifs present in *A. indica* and in *A. evenia* IRLF6945 ESTs (F. Cartieaux, unpublished data) were identified using the ESTtik pipeline developed by CIRAD (http://esttik.cirad.fr). Primers flanking the SSR motifs were designed using the Primer3 program (Table S6 B and C in File S1). RAPD and SSR markers were tested for PCR amplification using genomic DNA from *A. evenia* accessions as templates, as performed in Arrighi et al. [19]. PCR products were size-separated by standard horizontal electrophoresis, in 3.5% agarose gel for SSR markers and 1.5% agarose gel for RAPD, and visualized after staining with ethidium bromide. The gel images were recorded by use of a Vilber Lourmat transilluminator-camera system with the Infinity-Capt software (Vilbert Lourmat, Marne-la-Vallée, France).

The *A. evenia* EST library (F. Cartieaux, unpublished data) was used to identify single nuclear genes : *CYP1*, *eiF1a*, *Sucrose Synthase*, *SUI1* genes, a gene coding for a putative 2OG-Fe(II) oxygenase, and a legume-specific gene homolog to Glyma07g16420 and Glyma18g37410 identified in *Glycine max*. Corresponding primers were designed using the Primer3 program (Table S7 in File S1) and PCR reactions were performed using the Go Taq kit (Promega). The sequence comprising the ITS1–5.8S rDNA gene-ITS2 region was amplified with primers listed Table S7 in File S1. For PCR products with mixed sequences, they were cloned into pGEM-T Easy (Promega) following the manufacturer’s instructions. Individual transformed *E. coli* colonies were used as template for subsequent PCR amplification and sequencing.

The obtained sequences were deposited in GenBank. The references for all the sequences used in the phylogenetic analyses are listed Table S8 in File S1.

### Data Analysis

RAPD bands and SSR alleles obtained for the different *A. evenia* accessions were scored visually from gel images and converted into binary data by assigning 1 to presence or 0 to absence of a character or bands. They were then used to develop binary data matrix of *A. evenia* accessions. The DendroUPGMA software (http://genomes.urv.cat/UPGMA/) was used to produce for the different data sets a similarity matrix based the Dice similarity coefficient and a bootstrap of 100 replicates. The resulting data were further processed for cluster analysis using the un-weighted pair group average method with arithmetic mean algorithm (UPGMA) and the Neighbour program of the software package Phylip 3.5. The resulting clusters were presented as dendograms (tree phenogram).

For the phylogenetic analyses, the sequence of the 6 single nuclear genes was concatenated in order to obtain a well-resolved phylogeny. Concatenated single-gene sequences and ITS sequences were aligned using default settings in ClustalX [30] and were manually corrected in Genedoc v2.6 [31]. Phylogenetic reconstructions were performed using the MEGA v4 program [32] using the Neighbor Joining method and the Kimura-2 parameter model. Bootstrap (1,000 replicates) analyses were performed on data to place confidence estimates on groups contained in the most parsimonious trees generated. The data are presented as rooted trees using *A. denticulata* and *A. ciliata* as outgroups.

### DNA Content Measurements

DNA measurements were done by flow cytometry as presented in Arrighi et al. [19].

### Nomenclature

The electronic version of this article in Portable Document Format (PDF) in a work with an ISSN or ISBN will represent a published work according to the International Code of Nomenclature for algae, fungi, and plants, and hence the new names contained in the electronic publication of a PLOS ONE article are effectively published under that Code from the electronic edition alone, so there is no longer any need to provide printed copies.

In addition, new names contained in this work have been submitted to IPNI, from where they will be made available to the Global Names Index. The IPNI LSIDs can be resolved and the associated information viewed through any standard web browser by appending the LSID contained in this publication to the prefix http://ipni.org/. The online version of this work is archived and available from the following digital repositories: PubMed Central, LOCKSS.

## Supporting Information

Figure S1 **ITS nucleotide sequence alignment.** Nucleotide sequence comparison of the ITS sequences for representative *A. evenia* accessions using the Multalin software (v 5.4.1) (F. Corpet, INRA).
(TIF)

Figure S2 **Crossing barrier between the** ***evenia*** **and** ***serrulata*** **groups. A,** Seed morphology observed in the parent lines Bahia (CIAT8232) and Goias (IRFL6945) and the F1 seed obtained after manual crossings. Scale bars = 2.5 mm. **B,** Analysis of flower structure in both the Goias parent line (IRFL6945), Bahia parent line (CIAT8232) and the F1 progeny. (a) Front view of the flowers entire, (b) detailed view of stamens and pistil (arrow) in dissected flowers. Note that in both cases, dehisced anthers with liberated pollen face the stigma. Scale bar = 7 mm. **C,** Analysis of allele distributions for three polymorphic markers *Ai*SSR20 (a), *Ai*SSR36 (b) and *Ai*SSR38 (c) in four F2 plants from the inter-group crossing Goias ♂ (IRFL6945) × Bahia ♀ (CIAT8232).
(TIF)

File S1 **Supporting information.** Table S1. Phenotypic data of *Aeschynomene evenia* accessions. Table S2. List of discriminating molecular markers used for genotyping. Table S3. Similarity matrix calculated with DICE coefficient using 23 RAPD markers. Table S4. Distance matrix calculated with DICE coefficient using 82 SSR markers. Table S5. Nucleotidic polymorphism estimated with the comparison of the 2300 pb cumulated coding sequences. Table S6. List of molecular markers used for genotyping and their primer sequences. Table S7. Genes sequenced for the phylogenetic analysis. Table S8. GenBank numbers for the sequences used in the phylogenetic analyses.
(DOCX)
