# Supplementary figures and images for: Genotype Delimitation in the Nod-Independent Model Legume Aeschynomene evenia
Source: PLoS One. 2013 May 23;8(5):e63836. doi: 10.1371/journal.pone.0063836 (PMC3662760; doi:10.1371/journal.pone.0063836)

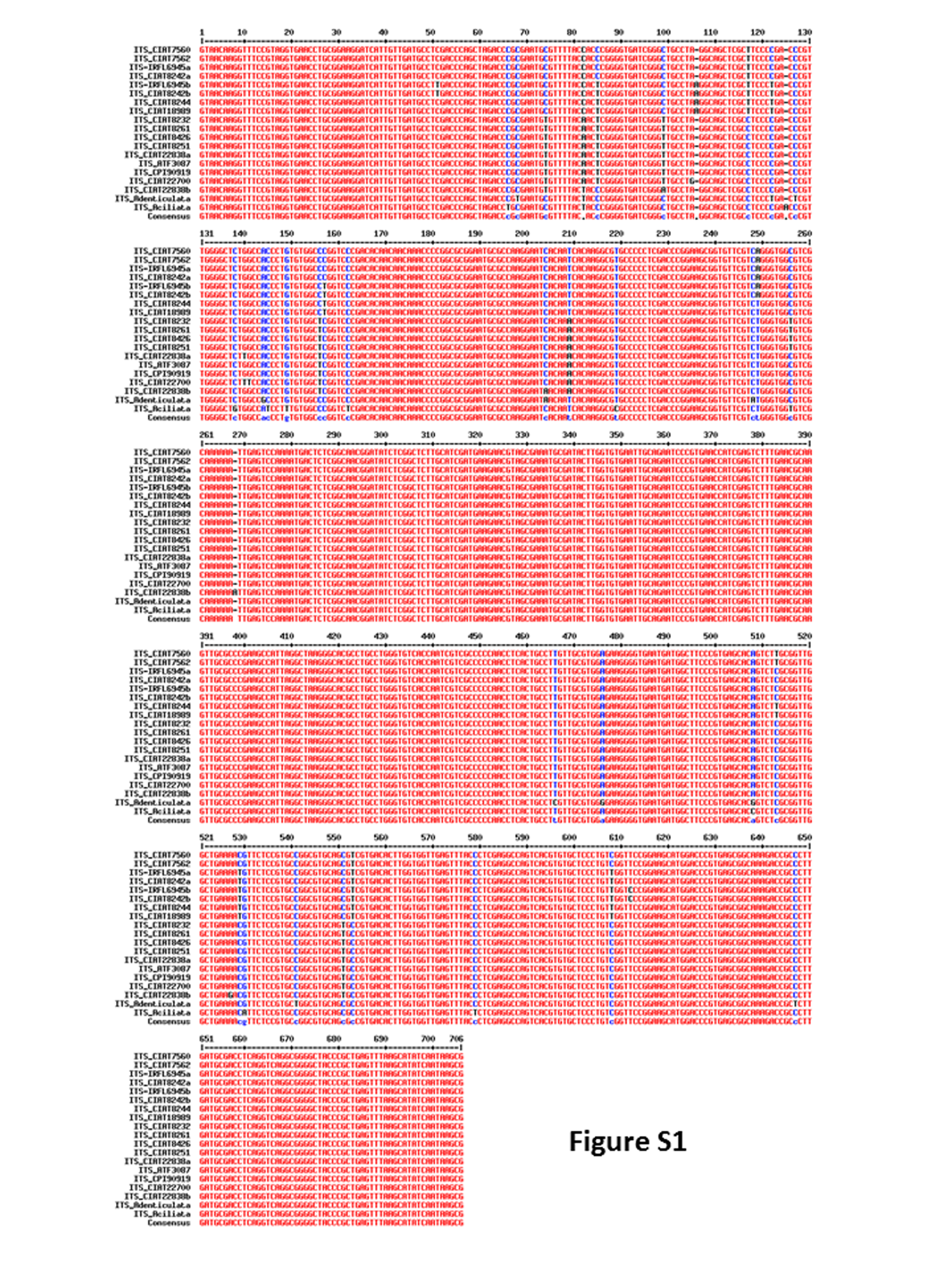

Supplement: Figure S1 — ITS nucleotide sequence alignment. Nucleotide sequence comparison of the ITS sequences for representative A. evenia accessions using the Multalin software (v 5.4.1) (F. Corpet, INRA). (TIF) [file pone.0063836.s001.tif]

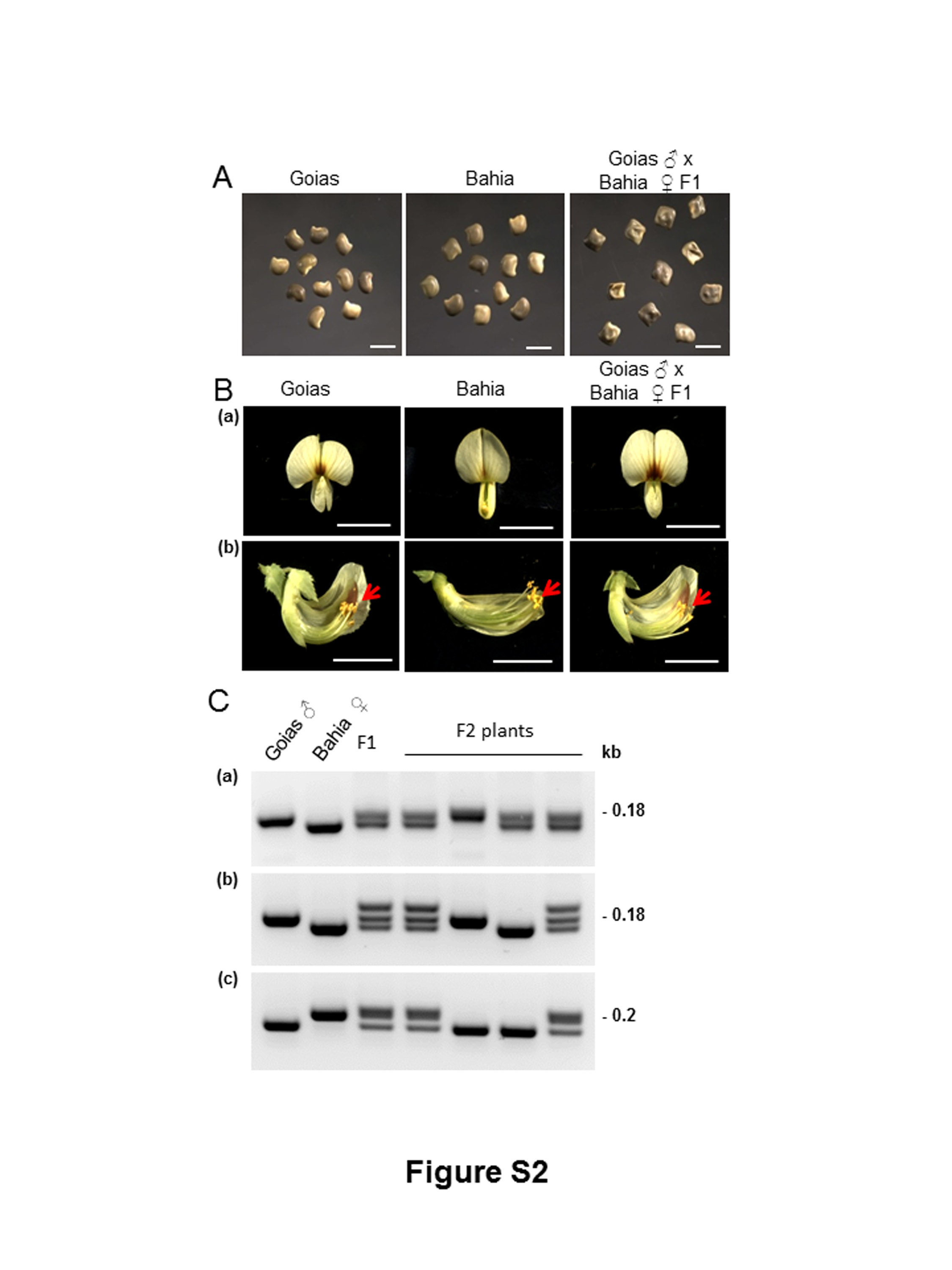

Supplement: Figure S2 — Crossing barrier between the evenia and serrulata groups. A, Seed morphology observed in the parent lines Bahia (CIAT8232) and Goias (IRFL6945) and the F1 seed obtained after manual crossings. Scale bars = 2.5 mm. B, Analysis of flower structure in both the Goias parent line (IRFL6945), Bahia parent line (CIAT8232) and the F1 progeny. (a) Front view of the flowers entire, (b) detailed view of stamens and pistil (arrow) in dissected flowers. Note that in both cases, dehisced anthers with liberated pollen face the stigma. Scale bar = 7 mm. C, Analysis of allele distributions for three polymorphic markers AiSSR20 (a), AiSSR36 (b) and AiSSR38 (c) in four F2 plants from the inter-group crossing Goias ♂ (IRFL6945) × Bahia ♀ (CIAT8232). (TIF) [file pone.0063836.s002.tif]
