# Supplementary material for: Genotype Delimitation in the Nod-Independent Model Legume Aeschynomene evenia
Source: PLoS One. 2013 May 23;8(5):e63836. doi: 10.1371/journal.pone.0063836 (PMC3662760; doi:10.1371/journal.pone.0063836)
Supplement: File S1 — Supporting information. Table S1. Phenotypic data of Aeschynomene evenia accessions. Table S2. List of discriminating molecular markers used for genotyping. Table S3. Similarity matrix calculated with DICE coefficient using 23 RAPD markers. Table S4. Distance matrix calculated with DICE coefficient using 82 SSR markers. Table S5. Nucleotidic polymorphism estimated with the comparison of the 2300 pb cumulated coding sequences. Table S6. List of molecular markers used for genotyping and their primer sequences. Table S7. Genes sequenced for the phylogenetic analysis. Table S8. GenBank numbers for the sequences used in the phylogenetic analyses. (DOCX) [file pone.0063836.s003.docx]

| **Table S1**: Phenotypic data of *Aeschynomene evenia* accessions | | | | | |  |  |  |  |  |
| --- | --- | --- | --- | --- | --- | --- | --- | --- | --- | --- |
|  |  |  |  |  |  |  |  |  |  |  |
| Group | Subroup | Accession | Plant habit | | Ramification | Stem | Leaf green color | Leaflet | n leaflets / leaf |  |
| *serrulata* | Goias | CIAT 7560 | robust | | down branched | glandular | dark | denticulate | 24.1 ± 2.7 |  |
|  |  | CIAT 7562 | robust | | down branched | glandular | dark | denticulate | 24.4 ± 2.9 |  |
|  |  | IRFL 6945 | robust | | down branched | glandular | dark | denticulate | 25.5 ± 3.2 |  |
|  | Alagoas | CIAT 8242 | robust but smaller | | down branched | glandular | dark | denticulate | 23.4 ± 2.9 |  |
|  |  | CIAT 8244 | robust but smaller | | down branched | glandular | dark | denticulate | 24.9 ± 3.6 |  |
|  |  | CIAT 18989 | robust but smaller | | down branched | glandular | dark | denticulate | 23.5 ± 2.7 |  |
| *evenia* | Bahia | CIAT 8232 | robust | | up ramified | glabrous | bright | few cilia | 24.1 ± 2.4 |  |
|  |  | CIAT 8261 | robust | | up ramified | glabrous | bright | few cilia | 21.4 ± 2.7 |  |
|  | Paraiba | CIAT 8426 | robust | | one axis | glabrous | bright | entire | 24.0 ± 0.7 |  |
|  |  | CIAT 8251 | robust | | one axis | glabrous | bright | entire | 26.0 ± 1.7 |  |
|  | Mbao | CIAT22700 | slender | | one axis | glabrous | bright | entire | 25.7 ± 2.7 |  |
|  |  | CIAT 22838 | robust | | one axis | glabrous | bright | entire | 26.1 ± 2.3 |  |
|  | Salta | ATF 3087 | slender | | intermediate | glabrous | bright | entire | 23.9 ± 2.9 |  |
|  |  | CPI 90919 | slender | | intermediate | glabrous | bright | entire | 23.1 ± 2.2 |  |
|  |  |  |  |  |  |  |  |  |  |  |
| **Table S1** : following | | |  |  |  |  |  |  |  |  |
|  |  |  |  |  |  |  |  |  |  |  |
| Group | Subgroup | Accession | Flower type | Flower length (mm) | Flower width (mm) | Stamen color | Pod shape (margins) | Pod epidermis | n seeds / pod | Seed size (mm) |
| *serrulata* | Goias | CIAT 7560 | *serrulata* | 8.5 ± 0.5 | 7.6 ± 0.5 | bright | crenate | trichomes | 7.3 ± 0.7 | 2.7 ± 0.1 |
|  |  | CIAT 7562 | *serrulata* | 8.6 ± 0.5 | 7.7 ± 0.5 | bright | crenate | trichomes | 7.4 ± 0,7 | 2.5 ± 0.1 |
|  |  | IRFL 6945 | *serrulata* | 9 ± 0.4 | 8.6 ± 0.3 | bright | crenate | trichomes | 7.6 ± 1.0 | 2.2 ± 0.2 |
|  | Alagoas | CIAT 8242 | *serrulata* | 7.7 ± 0.3 | 5.7 ± 0.3 | bright | crenate | trichomes | 6.2 ± 0.8 | 1.9 ± 0.1 |
|  |  | CIAT 8244 | *serrulata* | 7.5 ± 0.4 | 5.8 ± 0.3 | bright | crenate | trichomes | 6.0 ± 1.3 | 2.2 ± 0.1 |
|  |  | CIAT 18989 | *serrulata* | 7.5 ± 0.4 | 5.8 ± 0.3 | bright | crenate | trichomes | 6.0 ± 0.8 | 2.2 ± 0.1 |
| *evenia* | Bahia | CIAT 8232 | *evenia* | 9.7 ± 0.5 | 5.3 ± 0.2 | bright | subentire | ciliate | 8.6 ± 0.9 | 2.1 ± 0.2 |
|  |  | CIAT 8261 | *evenia* | 9.7 ± 0.5 | 5.3 ± 0.3 | bright | subentire | ciliate | 10.1 ± 1.4 | 2.2 ± 0.1 |
|  | Paraiba | CIAT 8426 | *evenia* | 9.8 ± 0.3 | 6.4 ± 0.4 | bright | subentire | ciliate | 8.6 ± 1.4 | 2.5 ± 0.1 |
|  |  | CIAT 8251 | *evenia* | 9.6 ± 0.4 | 5.7 ± 0.4 | bright | subentire | ciliate | 10.1 ± 1.0 | 2.4 ± 0.1 |
|  | Mbao | CIAT22700 | *evenia* | 9.8 ± 0.5 | 5.5 ± 0.4 | dark | subentire | glabrous | 8.0 ± 0.9 | 2.5 ± 0.1 |
|  |  | CIAT 22838 | *evenia* | 10.1 ± 0.4 | 6.7 ± 0.5 | dark | subentire | glabrous | 8.2 ± 1.0 | 3.0 ± 0.1 |
|  | Salta | ATF 3087 | *evenia* | 8.0 ± 0.4 | 4.5 ± 0.3 | bright | subentire | glabrous | 8.7 ± 1.7 | 2.4 ± 0.1 |
|  |  | CPI 90919 | *evenia* | 7.2 ± 0.4 | 5.2 ± 0.4 | bright | subentire | glabrous | 9.7 ± 1.8 | 2.4 ± 0.1 |

| **Table S2**: List of discriminating molecular markers used for genotyping | |
| --- | --- |
|  |  |
| A-RAPD markers with specific bands or specificly absent bands (into brackets) | |
| Category | RAPD |
| RAPD specifying the *serrulata* accessions | all |
| RAPD specifying the genotype Goias | OPAB-07, B06, B10, L14 |
| RAPD specifying the genotype Alagoas | OPA-11, OPAB-11, B6, G10, L03, L14 |
| RAPD specifying the *evenia* accessions | all |
| RAPD specifying the genotype Bahia | OPA-13, B06, B10, L12, L14 |
| RAPD specifying the genotype Paraiba | OPD-19, B06 (OPC-01, OPD-19, L17) |
| RAPD specifying the genotype Mbao | (B06, L14) |
| RAPD specifying the genotype Salto | OPAB-11, G17, M07 |
|  |  |
| B-*Ai*SSR markers |  |
| Category | *Ai*SSRs |
| Differential size between *evenia* and *serrulata* accessions | *Ai*SSR 2, *Ai*SSR 4, *Ai*SSR 5, *Ai*SSR 6, *Ai*SSR 8, *Ai*SSR 13, *Ai*SSR 43, *Ai*SSR 45, *Ai*SSR 51, *Ai*SSR 54, *Ai*SSR 62 |
| SSR markers amplified only in the *evenia* accessions | *Ai*SSR 31, *Ai*SSR 34 |
| SSR markers amplified only in the *serrulata* accessions | *Ai*SSR 16, *Ai*SSR 21, *Ai*SSR 26, |
|  |  |
| C-*Ae*SSR markers |  |
| Category | *Ae*SSRs |
| Differential size between *evenia* and *serrulata* accessions | *Ae*SSR5, *Ae*SSR7, *Ae*SSR20, *Ae*SSR21, *Ae*SSR28, *Ae*SSR32, *Ae*SSR35, *Ae*SSR42, *Ae*SSR46, *Ae*SSR47, *Ae*SSR48, *Ae*SSR56, *Ae*SSR59 |
| SSR markers amplified only in the *serrulata* accessions | *Ae*SSR 1, *Ae*SSR 2, *Ae*SSR 10, *Ae*SSR 14, *Ae*SSR 15, *Ae*SSR 16, *Ae*SSR 17, *Ae*SSR 23, *Ae*SSR 24, *Ae*SSR 25,*Ae*SSR 29, *Ae*SSR 31, *Ae*SSR 34, *Ae*SSR 38, *Ae*SSR 39, *Ae*SSR 40, *Ae*SSR 45, *Ae*SSR 52, *Ae*SSR 58 |

| **Table S3**: Similarity matrix calculated with DICE coefficient using 23 RAPD markers | | | | | | | | | | | | |  |  |  |
| --- | --- | --- | --- | --- | --- | --- | --- | --- | --- | --- | --- | --- | --- | --- | --- |
|  |  |  |  |  |  |  |  |  |  |  |  |  |  |  |  |
|  |  | *serrulata* group | | | | | | *evenia* group | | | | | | | |
|  |  | Goias | | | Alagoas | | | Bahia | | Paraiba | | Mbao | | Salta | |
|  |  | CIAT 7560 | CIAT 7562 | IRFL 6945 | CIAT 8242 | CIAT 8244 | CIAT 18989 | CIAT 8232 | CIAT 8261 | CIAT 8426 | CIAT 8251 | CIAT 22700 | CIAT 22838 | ATF 3087 | CPI 90919 |
| Goias | CIAT7560 | 100% | 99% | 98% | 69% | 67% | 84% | 15% | 15% | 15% | 16% | 14% | 12% | 13% | 15% |
|  | CIAT7562 |  | 100% | 97% | 69% | 67% | 85% | 15% | 15% | 16% | 16% | 14% | 12% | 13% | 15% |
|  | IRFL6945 |  |  | 100% | 68% | 66% | 84% | 13% | 13% | 14% | 14% | 12% | 9% | 11% | 13% |
| Alagoas | CIAT8242 |  |  |  | 100% | 95% | 85% | 15% | 15% | 18% | 18% | 14% | 12% | 15% | 15% |
|  | CIAT8244 |  |  |  |  | 100% | 80% | 16% | 16% | 18% | 19% | 14% | 12% | 16% | 16% |
|  | CIAT18989 |  |  |  |  |  | 100% | 17% | 17% | 19% | 20% | 16% | 14% | 17% | 17% |
| Bahia | CIAT8232 |  |  |  |  |  |  | 100% | 100% | 74% | 71% | 77% | 78% | 78% | 79% |
|  | CIAT8261 |  |  |  |  |  |  |  | 100% | 74% | 71% | 77% | 78% | 78% | 79% |
| Paraiba | CIAT8426 |  |  |  |  |  |  |  |  | 100% | 98% | 81% | 82% | 82% | 76% |
|  | CIAT8251 |  |  |  |  |  |  |  |  |  | 100% | 81% | 82% | 80% | 73% |
| Mbao | CIAT22700 |  |  |  |  |  |  |  |  |  |  | 100% | 96% | 83% | 82% |
|  | CIAT22838 |  |  |  |  |  |  |  |  |  |  |  | 100% | 84% | 85% |
| Salta | ATF3087 |  |  |  |  |  |  |  |  |  |  |  |  | 100% | 94% |
|  | CPI90919 |  |  |  |  |  |  |  |  |  |  |  |  |  | 100% |

| **Table S4**: Distance matrix calculated with DICE coefficient using 82 SSR markers | | | | | | | | | | | | |  |  |  |
| --- | --- | --- | --- | --- | --- | --- | --- | --- | --- | --- | --- | --- | --- | --- | --- |
|  |  |  |  |  |  |  |  |  |  |  |  |  |  |  |  |
|  |  | *serrulata* group | | | | | | *evenia* group | | | | | | | |
|  |  | Goias | | | Alagoas | | | Bahia | | Paraiba | | Mbao | | Salta | |
|  |  | CIAT 7560 | CIAT 7562 | IRFL 6945 | CIAT 8242 | CIAT 8244 | CIAT 18989 | CIAT 8232 | CIAT 8261 | CIAT 8426 | CIAT 8251 | CIAT 22700 | CIAT 22838 | ATF 3087 | CPI 90919 |
| Goias | CIAT7560 | 0 | 11% | 19% | 31% | 37% | 22% | 70% | 69% | 65% | 69% | 67% | 69% | 72% | 71% |
|  | CIAT7562 |  | 0 | 20% | 32% | 34% | 26% | 74% | 73% | 70% | 70% | 70% | 74% | 76% | 74% |
|  | IRFL6945 |  |  | 0 | 35% | 37% | 27% | 74% | 72% | 69% | 70% | 72% | 74% | 75% | 73% |
| Alagoas | CIAT8242 |  |  |  | 0 | 15% | 25% | 76% | 75% | 72% | 73% | 72% | 71% | 77% | 76% |
|  | CIAT8244 |  |  |  |  | 0 | 25% | 74% | 73% | 70% | 69% | 70% | 72% | 75% | 73% |
|  | CIAT18989 |  |  |  |  |  | 0 | 69% | 70% | 65% | 64% | 65% | 66% | 71% | 67% |
| Bahia | CIAT8232 |  |  |  |  |  |  | 0 | 13% | 25% | 28% | 30% | 32% | 28% | 27% |
|  | CIAT8261 |  |  |  |  |  |  |  | 0 | 25% | 25% | 30% | 33% | 26% | 24% |
| Paraiba | CIAT8426 |  |  |  |  |  |  |  |  | 0 | 18% | 20% | 23% | 26% | 26% |
|  | CIAT8251 |  |  |  |  |  |  |  |  |  | 0 | 20% | 23% | 19% | 24% |
| Mbao | CIAT22700 |  |  |  |  |  |  |  |  |  |  | 0 | 17% | 24% | 19% |
|  | CIAT22838 |  |  |  |  |  |  |  |  |  |  |  | 0 | 23% | 29% |
| Salta | ATF3087 |  |  |  |  |  |  |  |  |  |  |  |  | 0% | 20% |
|  | CPI 90919 |  |  |  |  |  |  |  |  |  |  |  |  |  | 0% |
|  |  |  |  |  |  |  |  |  |  |  |  |  |  |  |  |
| Polymorphism level between *serrulata* accessions was determined using 82 *Ai*SSR and *Ae*SSR markers. Because 19 *Ae*SSRs could not be amplified for the *evenia* accessions, they were not taken into account to estimate polymorphism with these accessions. | | | | | | | | | | | | | | | |

| **Table S5**: Nucleotidic polymorphism estimated with the comparison of the 2300 pb cumulated coding sequences | | | | | | | | | | | | | | | |
| --- | --- | --- | --- | --- | --- | --- | --- | --- | --- | --- | --- | --- | --- | --- | --- |
|  |  |  |  |  |  |  |  |  |  |  |  |  |  |  |  |
|  |  | *serrulata* group | | | | | | *evenia* group | | | | | | | |
|  |  | Goias | | | Alagoas | | | Bahia | | Paraiba | | Mbao | | Salta | |
|  |  | CIAT 7560 | CIAT 7562 | IRFL 6945 | CIAT 8242 | CIAT 8244 | CIAT 18989 | CIAT 8232 | CIAT 8261 | CIAT 8426 | CIAT 8251 | CIAT 22700 | CIAT 22838 | ATF 3087 | CPI 90919 |
| Goias | CIAT7560 | - | 1 | 0 | 1 | 2 | 1 | 27 | 28 | 27 | 28 | 27 | 27 | 28 | 28 |
|  | CIAT7562 |  | - | 0 | 1 | 1 | 0 | 28 | 29 | 28 | 29 | 28 | 28 | 29 | 29 |
|  | IRFL6945 |  |  | - | 1 | 2 | 1 | 27 | 28 | 27 | 28 | 27 | 27 | 28 | 27 |
| Alagoas | CIAT8242 |  |  |  | - | 1 | 2 | 28 | 29 | 28 | 29 | 28 | 28 | 29 | 29 |
|  | CIAT8244 |  |  |  |  | - | 1 | 29 | 30 | 29 | 30 | 29 | 29 | 30 | 30 |
|  | CIAT18989 |  |  |  |  |  | - | 28 | 29 | 28 | 29 | 28 | 28 | 29 | 29 |
| Bahia | CIAT8232 |  |  |  |  |  |  | - | 1 | 4 | 5 | 4 | 5 | 7 | 5 |
|  | CIAT8261 |  |  |  |  |  |  |  | - | 5 | 4 | 5 | 6 | 6 | 6 |
| Paraiba | CIAT8426 |  |  |  |  |  |  |  |  | - | 1 | 2 | 3 | 5 | 3 |
|  | CIAT8251 |  |  |  |  |  |  |  |  |  | - | 3 | 4 | 4 | 4 |
| Mbao | CIAT22700 |  |  |  |  |  |  |  |  |  |  | - | 3 | 5 | 3 |
|  | CIAT22838 |  |  |  |  |  |  |  |  |  |  |  | - | 6 | 4 |
| Salta | ATF3087 |  |  |  |  |  |  |  |  |  |  |  |  | - | 6 |
|  | CPI 90919 |  |  |  |  |  |  |  |  |  |  |  |  |  | - |
|  |  |  |  |  |  |  |  |  |  |  |  |  |  |  |  |
| Polymorphism level corresponds to the number of different nucleotides or motif repeats in the cumulated coding sequences of the six nuclear genes analysed, between two subgroups. | | | | | | | | | | | | | | | |

| **Table S6**: List of molecular markers used for genotyping and their primer sequences | | | | |
| --- | --- | --- | --- | --- |
|  |  |  |  |  |
| A-RAPD markers | |  |  |  |
| Name | Primer (5'-3') |  |  |  |
| OPA-10 | GTGATCGCAG |  |  |  |
| OPA-11 | CAATCGCCGT |  |  |  |
| OPA-13 | CAGCACCCAC |  |  |  |
| OPC-01 | TTCGAGCCAG |  |  |  |
| OPD-11 | AGCGCCATTG |  |  |  |
| OPD-19 | GGGGTGACGA |  |  |  |
| OPP-09 | GTGGTCCGCA |  |  |  |
| OPP-16 | CCAAGCTGCC |  |  |  |
| OPAB-07* | GTAAACCGCC |  |  |  |
| OPAB-11* | GTGCGCAATG |  |  |  |
| B01 | GTTTCGCTCC |  |  |  |
| B06 | TGCTCTGCCC |  |  |  |
| B10 | CTGCTGGGAC |  |  |  |
| G03* | GAGCCCTCCA |  |  |  |
| G10* | AGGGCCGTCT |  |  |  |
| G17* | ACGACCGACA |  |  |  |
| L02 | TGGGCGTCAA |  |  |  |
| L03* | CCAGCAGCTT |  |  |  |
| L12* | GGGCGGTACT |  |  |  |
| L14* | GTGACAGGCT |  |  |  |
| L17* | AGCCTGAGCC |  |  |  |
| M07* | CCGTGACTCA |  |  |  |
| M10 | TCTGGCGCAC |  |  |  |

| B-SSR markers derived from *Aeschynomene indica* | | |  |
| --- | --- | --- | --- |
| Name | Repeat motif | Forward primer (5'-3') | Reverse primer (5'-3') |
| *Ai*SSR2* | (AAT)_20_ | TTGAAAGATCCCTGCCAAAT | TGGTGTTCTTGAATATGGTGCT |
| *Ai*SSR3* | (CT)_10_(CA)_11_ | CAAACACACACCTCCCTTCA | GCTGAACCCGAAGAGCTTTA |
| *Ai*SSR4* | (GA)_16_ | TCAACAGAAAAATGGCCACA | TCAAGATGACCACCACCTGA |
| *Ai*SSR5* | (AG)_16_ | TGACTGTGGGAAACGAACAA | GAAGAAAAGGGGCTTCTTGG |
| *Ai*SSR6* | (TC)_19_ | TGATTTCACTTGGCTCATCG | GAGAGATCGCTGCAGAAAGG |
| *Ai*SSR7* | (TAT)_15_ | GCGAGCGAACTGAACAAAGT | TCCCCAAAAGACCAGCATAG |
| *Ai*SSR8* | (AG)_12_ | AACCACAGAGAAAAGTAGAAGACGA | CCTCGACGAGCACACTATCA |
| *Ai*SSR13* | (AG)_12_ | GAACGAATACACTCCAAAGAAGAAA | ATTCAACACTGCAGCAACCA |
| *Ai*SSR14 | (AG)_14_ | CACTGGTGGTATTGCGTTTG | TGCTCATGTCCAAGAAGCAC |
| *Ai*SSR15* | (AG)_11_ | GCTGCGTGAGTGCGTAACTA | TGTTCGTGTCGAAGGTTGAG |
| *Ai*SSR16 | (TA)_11_ | TCCAGATTCTGAACCATTTTCC | TGCCATTTAACTTTTCTGAATTTG |
| *Ai*SSR17 | (CT)_11_ | AAGAGCTCCCACCAAACAAA | CGTTTAATGTCTTCATCCACAAA |
| *Ai*SSR18* | (TC)_11_ | CGGAGCCATGATCTCTCAGT | GAACACGTCGAAACAATGGA |
| *Ai*SSR20* | (AT)_11_ | CTGCGAAATGTGCTGCTAAA | GCCATAAGTACAAGAGGAAGATGA |
| *Ai*SSR21 | (AT)_11_ | CAGAGATAACTTCGGGACCAA | GACCAACCAAAACCGACAAC |
| *Ai*SSR23* | (CT)_12_ | GCTGGGTTGTGTGAACACTCT | GGTGTAGTTTCCGCGTTGAT |
| *Ai*SSR24* | (AC)_6_X_15_(AG)_13_ | GATTCTGGGGGAGACACAA | CCTTCCACCTCGATCACCT |
| *Ai*SSR26* | (AG)_12_ | TCAGCTCAAATATCTCAAACCCTA | AAACTGCTACGGAGGCAGAG |
| *Ai*SSR29 | (AG)_9_ | GGATGCAGAAGCTGAAACTG | CTAGCCCAATCCAGTGCTTC |
| *Ai*SSR31 | (TA)_9_ | CCACAGAGGATACAAGCAAGAA | GGACAATCTTTGCAGTGAAGC |
| *Ai*SSR34 | (TC)_9_ | CACTCGCACCTTATTTTCCAA | GGAAGGGAAAGGAGAGGTTG |
| *Ai*SSR36* | (AC)_9_ | ACAGTTTGGTGTGTCGATGC | CCAGGAGGACTCATTTTCCA |
| *Ai*SSR37* | (CT)_9_ | CGCAAGGGGTTTCAACTAGA | GAAGGAAGAAGGACGACACC |
| *Ai*SSR39* | (GA)_9_ | CAGTTCGTTGGTGAGAAGCA | AGCGAGGACCCTCTGTATGA |
| *Ai*SSR43* | (ATG)_9_ | GCTCAGGAACTCTCCAATGC | TCCCTTCCTGAAGATGCTTG |
| *Ai*SSR45 | (AG)_16_ | TGACTGTGGGAAACGAACAA | GAAGAAAAGGGGCTTCTTGG |
| *Ai*SSR50 | (TC)_19_ | AAGAATCCCCGGAATCAGTT | GAGAGATCGCTGCAGAAAGG |
| *Ai*SSR51 | (AT)_11_ | CTGCGAAATGTGCTGCTAAA | GCCATAAGTACAAGAGGAAGATGA |
| *Ai*SSR53 | (TA)_9_ | ACGAAGCCTTCACCTCAAAA | TTGTGTCCCATGAAACAAGC |
| *Ai*SSR54 | (TA)_11_ | TGCTGAAATGGGTGTTGAAG | AGATGTGGTTGTGGAGCATAA |
| *Ai*SSR57 | (CT)_12_ | CCTCGCTTCATTCTTTTTCG | AACAACAACGCTGGATCCTT |
| *Ai*SSR62 | (AG)_10_ | CCCTTTTTCCTTCGGTTTTC | TCAGCTTTCGCTAACTGATCC |
| *Ai*SSR63 | (AG)_9_ | ACGCCATTAACGACCTCAAC | GCGACGAATGAGTTCAGACA |

| C-SSR markers derived from *Aeschynomene evenia* | |  |  |
| --- | --- | --- | --- |
| Name | Repeat motif | Forward primer (5'-3') | Reverse primer (5'-3') |
| *Ae*SSR1 | (TC)_8_ | TTGACCACTTCACTTCTCAATTAAA | CCAGCACTGCATAGCACAAT |
| *Ae*SSR2 | (CT)_9_ | CACTCCTCACTCCCCCACT | CGTCATGAACACCGTGTAGG |
| *Ae*SSR3 | (GA)_8_ | TGATGGTAGCAATCAATGAAAAA | AAGAGAGAGCAGCCACAAGC |
| *Ae*SSR4 | (CT)_8_ | GCCTCACTTGGGGTTTTCTT | ATCTCGGAACAACGAGCATC |
| *Ae*SSR5 | (CT)_13_ | CTGACTGGAAGGAGCGAAAA | CGAGGACCGAGTCCATAAGA |
| *Ae*SSR7 | (AT)_8_ | AGCATGGACGTTGGGTAGAC | CCCACCCCCAGGCTATACTA |
| *Ae*SSR9 | (CT)_8_ | CCGAATCCTCGTTCTCAAAC | GAAGATTCCCAGACCCGAAT |
| *Ae*SSR10 | (AT)_9_ | CGTCCCGATAACATCCTCCT | ATCTCCGATCCATTTGGTAAT |
| *Ae*SSR11 | (GT)_8_ | ACAAAGCTGGTTTCGTTGCT | GAGGGAGAATTTGAGAATGAGG |
| *Ae*SSR12 | (TC)_11_ | CCTAATCCCACACCGCAGTA | GAAGCAAGGTTTGGTGGGTA |
| *Ae*SSR14 | (CA)_12_ | CTCTCCGATCCATCATCACC | GCTGAGAGCCACAATCTGGT |
| *Ae*SSR15 | (TG)_11_ | GGCCCTTTGTGGGATTTAGA | CTGTAATGGTGGTGGTGGTG |
| *Ae*SSR16 | (GA)_10_ | GGTGGGGTGGTCCAAGTAG | GTTGTGGAGGTGGAGCAGAT |
| *Ae*SSR17 | (CT)_8_ | TGCAGTTCAGTGCCTCTCTC | CCTCAGAAACCGAAACCAAA |
| *Ae*SSR18 | (TC)_10_ | CCAGAGAGGCCTTTCCTCTT | GATTTGGAGGTCGATTGTGG |
| *Ae*SSR19 | (GA)_11_ | TGCAACTGGCAAAACCATAA | GGAAAAACGAACAAGGAGCA |
| *Ae*SSR20 | (TC)_11_ | TCCGCATGGAGGAGTAAAAG | AAAGGGGGATAGCATAGGATG |
| *Ae*SSR21 | (AG)_10_ | GAAAGGAACGTGTTGGAAGC | GAAGGCAACTGCGATTGATT |
| *Ae*SSR23 | (AT)_9_ | AAACCAAACCAAACCAGCAG | CTGAGTTGTTGCCTCGGAAT |
| *Ae*SSR24 | (TA)_10_ | CGATAAGCCCGTTCATTTGT | TGAAGCGTGCTAAGAATTGC |
| *Ae*SSR25 | (CT)_12_ | AACAGTTGCGTTTTGCCATC | GGTGGAGATGAGCTTTCCTG |
| *Ae*SSR26 | (CT)_8_ | ACATCGCTGTCTTTCGGTCT | AGAAGAGGGGTTTGGCTCTC |
| *Ae*SSR28 | (AT)_12_ | ACAACACAGAAACAAAGAAAGAAAGA | GGCTCTGAACCTTCCTCACC |
| *Ae*SSR29 | (TA)_8_ | CGGCAGAAGCCACTCTTAAC | CTTGTTCCCCAAAACCATTG |
| *Ae*SSR30 | (CT)_9_ | AATCACGTTCCCACAGATCC | GTTGTTGGTGGAGCTGGATT |
| *Ae*SSR31 | (CT)_12_ | TCCATTCATTCCTTCCTTCG | TTGATGTGGGGAGTGAGTGA |
| *Ae*SSR32 | (GA)_10_ | GTGTTTGAGAGGGGCACAG | TCCTCCCTCTCCATGAGTTG |
| *Ae*SSR33 | (CT)_10_ | GTCTCCCACTCTCACCAAGC | AGATGGCTGAATCTGCGTTT |
| *Ae*SSR34 | (TC)_8_ | CACCCTCCTTCAAACACCAT | GTCATCTCGGACTCGCTTGT |
| *Ae*SSR35 | (AG)_9_ | CACCAACAACACCTCCCTCT | AGAGCGTCCATCAGATCCTC |
| *Ae*SSR36 | (CT)_11_ | AGAAGGCGAAAGGGAAAAAG | CTAGGGTTTCAGTGGGAACG |
| *Ae*SSR38 | (CT)_9_ | GGGCCAACACACTTATCACC | TGGTGTTCCCAGAAAGGGTA |
| *Ae*SSR39 | (TA)_8_ | CCCAAAGAGGCAAACAAAAG | TCCCTCCTGAGAGCCAAATA |
| *Ae*SSR40 | (TC)_9_ | AAGACGATCCAGCAATGACC | GGGTTGTGGAAGTGGAAGAA |
| *Ae*SSR42 | (CT)_12_ | TAACCACCAAGCCAAACACA | GGAAAGGACGTTCAGAATCAA |
| *Ae*SSR43 | (TC)_10_ | TCAACCCATAACTACCGTCATTT | AGAAGGACCAAGCCTTCCAT |
| *Ae*SSR45 | (GA)_10_ | AGGGTTAGGGGATTAGAAGAGC | CCTCCTTCCTCCGTCAATCT |
| *Ae*SSR46 | (TC)_8_ | CCCCCAGAATTCTCAACCTT | TGATTTCGAGACCCATTTGA |
| *Ae*SSR47 | (AG)_9_ | CAAGTGTACGGAGGGAAGGA | CCTCCTTGGTGTGTTCTGGT |
| *Ae*SSR48 | (AG)_10_ | ATTTCTTGGTTGGCACGAAT | GACCAACCTGAAGAGCAAGC |
| *Ae*SSR49 | (CAA)_17_ | GGCGAGTTCCTCCTCTCTCT | TAATCGTGACCGTTGGATGA |
| *Ae*SSR50 | (CA)_9_ | GGTCTCCGTTGGGTTACACT | AGGCGCAGAAATGAGAAGAA |
| *Ae*SSR51 | (TC)_9_ | CACGTCACTTTTCCATCCAA | CCACCAAGTGGGCATTCTAA |
| *Ae*SSR52 | (TC)_8_ | AACCCTACCACCACCTACCC | GGTAGAGGGTGACCGGAAGT |
| *Ae*SSR53 | (TC)_8_ | TCCCTCTCCTTTCTTCAAACC | GGAGACGCCATTGTTTTGTT |
| *Ae*SSR55 | (TA)_8_ | CAGATTCAGCAGGGTGGTG | GTCCATGGTTTCAGCCTCAT |
| *Ae*SSR56 | (AT)_10_ | AGCGCCACAGATCCTCTCTA | GTTGGTTGATCTGGGGAAGA |
| *Ae*SSR57 | (TA)_8_ | GGAGGAGCTTGCAGTTGTTC | AAGCCCTGTTTGTTCTCTTGA |
| *Ae*SSR58 | (AG)_11_ | TCCATCCTCATCACACAAACA | CGTTGAGACCTGCAGCATTA |
| *Ae*SSR59 | (AT)_13_ | CAAATGGCAATGGTGTTCAG | GCCTTGCTACCTGCGTTATC |
| *Ae*SSR60 | (GA)_11_ | GGAGAAATTGTGCTGTGGAGA | ACCACATAGCACAAGAACAGAA |
|  |  |  |  |
| * : markers previously used in [19] | | |  |

| **Table S7:** Genes sequenced for the phylogenetic analysis | | | |  |  |
| --- | --- | --- | --- | --- | --- |
|  |  |  |  |  |  |
| Gene | Orthologs in *Glycine max* | *A. evenia* EST | Primers 5'-3' | PCR product |  |
| *SUI1* | Glyma18g11010 Glyma08g42860 | CL414Contig1 | F : AAATCCAGTTCCCCACACC R : CAGAAACCATGAATCTTGATCTG | 325 pb |  |
| *eiF1a* | Glyma19g44160 Glyma03g41560 | CL1312Contig1 | F : ATGCCGAAGAACAAGGGAAA R : ATCAATATCCTCATCCTCGAACTC | 429 pb |  |
| *CYP1* | Glyma12g02790 Glyma11g10480 | CL604Contig4 | F : GCGAACCCTAGAGTTTACTTCG R : GCAATCTGCGATCACAACC | 501 pb |  |
| Legume-specific gene | Glyma07g16420 Glyma18g37410 | CL2845Contig2 | F : CACCAAAAATGGAGGGTCTT R : TGAAAGAACAAACTTGATAGACTTGA | 346 pb |  |
| *Sucrose Synthase* | Glyma15g20180 Glyma09g08550 | CL150Contig1 | F : GAAGGACCGTAGCAAACCAA R : CCCAGTGAGAAGGATCAACC | 515 pb |  |
| Putative 2OG-Fe(II) oxygenase | Glyma08g09930 Glyma05g26950 | CL2690Contig1 | F : TCGGGTAATCCATCTCAACAG R : GCGCTTTACATGTTGCTTCA | 403 pb |  |
| *ITS* | - | - | F : GTCCACTGAACCCTTATCATTTAGAGG R : GCCGTTACTAAGGGAATCCTTGTTAG | 704 pb |  |
|  |  |  |  |  |  |
| *CYP1* corresponds to the cyclophylin 1, *eif1α* corresponds to the eukaryotic translation initiation factor 1A, *SUI1* is a translation factor and the legume-specific gene is a homolog to Glyma07g16420 and Glyma18g37410 identified in *Glycine max*. | | | | |  |
|  |  |  |  |  |  |

| **Table S8:** GenBank numbers for the sequences used in the phylogenetic analyses | | | | | | |  |  |
| --- | --- | --- | --- | --- | --- | --- | --- | --- |
|  |  |  |  |  |  |  |  |  |
| Species | Accession | *SUI1* | *eiF1a* | *CYP1* | Legume-specific gene | *Sucrose Synthase* | Putative 2OG-Fe(II) oxygenase | *ITS* |
| *A. evenia* | CIAT7560 | KC163273 | KC163221 | KC163208 | KC163234 | KC163260 | KC163247 | KC163286 |
|  | CIAT7562 | KC163274 | KC163222 | KC163209 | KC163235 | KC163261 | KC163248 | KC163287 |
|  | IRFL6945 | KC163275 | KC163223 | KC163210 | KC163236 | KC163262 | KC163249 | KC163288 KC163289 |
|  | CIAT8242 | KC163276 | KC163224 | KC163211 | KC163237 | KC163263 | KC163250 | KC163290 KC163291 |
|  | CIAT8244 | KC163277 | KC163225 | KC163212 | KC163238 | KC163264 | KC163251 | KC163292 |
|  | CIAT18989 | KC163278 | KC163226 | KC163213 | KC163239 | KC163265 | KC163252 | KC163293 |
|  | CIAT8232 | KC163279 | KC163227 | KC163214 | KC163240 | KC163266 | KC163253 | KC163294 |
|  | CIAT8261 | KC163280 | KC163228 | KC163215 | KC163241 | KC163267 | KC163254 | KC163295 |
|  | CIAT8426 | KC163281 | KC163229 | KC163216 | KC163242 | KC163268 | KC163255 | KC163296 |
|  | CIAT8251 | KC163282 | KC163230 | KC163217 | KC163243 | KC163269 | KC163256 | KC163297 |
|  | CIAT22700 | KC163283 | KC163231 | KC163218 | KC163244 | KC163270 | KC163257 | KC163298 |
|  | CIAT22838 | KC163284 | KC163232 | KC163219 | KC163245 | KC163271 | KC163258 | KC163299 KC163300 |
|  | ATF3087 | KC294561 | KC294549 | KC294546 | KC294552 | KC294558 | KC294555 | KC294564 |
|  | CPI90919 | KC294562 | KC294550 | KC294547 | KC294553 | KC294559 | KC294556 | KC294565 |
| *A. denticulata* | IRRI13003 | KC163285 | KC163233 | KC163220 | KC163245 | KC163272 | KC163259 | FM242626* |
| *A. ciliata* | IRRI13078 | KC294563 | KC294551 | KC294548 | KC294554 | KC294560 | KC294557 | FM242624* |
|  |  |  |  |  |  |  |  |  |
| *: Indicates sequences directly retrieved from Genbank. | | | | |  |  |  |  |
